# An Honest Confession

**Published:** 1875-08

**Authors:** 


An Honest Confession is Good for the Soul.—One of our subscribers, after expressions of satisfaction, remarks: "There is one objection to your Journal, in my estimation, viz: the facility by and through which nostrum vendors palm off their infernal stuff upon the profession; a very adroit scheme on their part. A physician seeing an advertisement in the newspapers, would not give it a second thought, but when presented to him in his medical journal he is apt to be misled. If ever the profession is circumvented by the proprietary scoundrels it will be through the medium of the medical journals. Now, I submit to you, doctor, should such things be?"

We heartily agree with the position of our correspondent upon the abstract principle involved. Medical journals, as the exponents of the higher sentiment of the profession, ought to be, like Cæsar's wife, not only pure, but above suspicion. There is unquestionably a power in the medical press, and medical editors ought to be held responsible that this power is used for the elevation and advancement of the ethical as well as the scientific interests of the great medical body they serve. Whilst the medical editor is set, for the time being, as a sentinel upon the watch tower, it must not be forgotten that he is not thereby exempted from the weaknesses and foibles which pertain to mortals; nor is he, in his final mortuary analysis, aught else than carbonic acid, water and ammonia, just as other men are found to be. On the other hand it is to be remembered that medical editors are doctors, and that doctors, as a class, are not altogether what they ought to be; *ergo*, medical editors, as a class, cannot be expected to be altogether what they ought to be. In hydraulics it has been found difficult to elevate a stream higher than the source from which its aqueous supplies are drawn, and in medical journalism it will always be difficult to advance the press much beyond the standard of the medical profession whose mouthpiece it is.

But to the immediate matter in point: In our experience as a journalist and leader of medical thought, we have been disposed to look to the older and more influential organs of the profession, which are issued from the great medical centers, for examples for our imitation and emulation. Judged by this standard we have not found ourselves in the past at all remiss in our selections for our advertising columns. The *London Lancet* (original copy) is probably unequaled by any other medical journal in the world in the ability of its editorial staff and the wide-spread diffusion of its influence, and yet we find regularly advertised in its columns various proprietary medicines of secret composition, and this with numerous certificates of wonderful cures. The same may be said of American journals of the greatest influence in a few instances. Upon the continent of Europe it is still worse. Whilst physicians and surgeons of eminence admit into hospitals under their charge notorious and arrant quacks to test the virtues of their nostrums upon the inmates, it will be difficult to keep them out of the medical journals.

We are, however, of the opinion that a proper degree of circumspection in this matter is incumbent upon the conductors of journals. We think that the composition of proprietary remedies advertised in medical journals ought to be given, and that the usual trappings and paraphernalia of nostrums and patent medicines should not be allowed upon bottles and packages thus advertised.

